# Erythrokeratodermia‐Cardiomyopathy Syndrome: Expanding the *DSP* Mutational Spectrum Beyond Proline Substitutions

**DOI:** 10.1111/pde.70048

**Published:** 2025-10-14

**Authors:** Sepideh Hamzehlou, Ryland D. Mortlock, Caroline Echeandia‐Francis, Jing Zhou, Chao Xing, Nnenna G. Agim, Keith A. Choate, Donald A. Glass

**Affiliations:** ^1^ Department of Dermatology University of Texas Southwestern Medical Center Dallas Texas USA; ^2^ Department of Dermatology Yale University, School of Medicine New Haven Connecticut USA; ^3^ Department of Genetics Yale University, School of Medicine New Haven Connecticut USA; ^4^ McDermott Center for Human Growth and Development University of Texas Southwestern Medical Center Dallas Texas USA; ^5^ Department of Bioinformatics University of Texas Southwestern Medical Center Dallas Texas USA; ^6^ Department of Population and Data Sciences University of Texas Southwestern Medical Center Dallas Texas USA; ^7^ North Dallas Dermatology Associates Dallas Texas USA; ^8^ Department of Pathology Yale University, School of Medicine New Haven Connecticut USA

## Abstract

Erythrokeratodermia cardiomyopathy (EKC) syndrome is a rare autosomal dominant disorder characterized by generalized erythrokeratoderma and progressive dilated cardiomyopathy, caused by pathogenic variants in the SR6 domain of desmoplakin (*DSP*). We report two cases of EKC with novel *de novo* missense *DSP* variants at phenylalanine position 590 (F590S and F590V), expanding the mutational spectrum beyond proline substitutions. Immunostaining demonstrated disrupted desmosomal protein localization. One patient showed significant clinical improvement with ustekinumab therapy. These findings underscore the need for early cardiac monitoring and support IL‐12/23p40 inhibition as a potential therapeutic strategy in EKC.

## 1 Introduction

Erythrokeratodermia cardiomyopathy (EKC) syndrome is a rare autosomal dominant disorder of generalized cutaneous erythrokeratoderma and progressive dilated cardiomyopathy. EKC syndrome is caused by damaging variants within the spectrin repeat 6 (SR6) domain of the *desmoplakin* (*DSP*) gene, which codes for desmoplakin, the most abundant protein in desmosomes. Desmosomes are intercellular junctions that provide strong cell‐to‐cell adhesion via anchoring intermediate filaments to desmosomal plaques [1]. Desmosomes are therefore particularly abundant in tissues such as the epidermis and myocardium that are continually subject to mechanical stress.

EKC was first described by Boyden et al. [2] in 2016, describing three children with heterozygous de novo missense variants in the SR6 domain of *DSP* resulting in proline substitutions (Q616P, H618P, and L622P). The authors concluded that these missense variants likely disrupted the native helical structure of the conserved spectrin repeat (SR) 6 domain, as proline is a strong α‐helix breaker. Additionally, there are two other EKC‐causing novel variants in the literature that report proline substitutions for native amino acids (L583P, S610P) [3, 4]. Here we report two cases of EKC due to de novo missense variants in the SR6 domain of *DSP* resulting in substitution of the native amino acid phenylalanine at position 590 for serine and valine.

## 2 Case Report

### 2.1 Index Case 1

**FIGURE 1:**
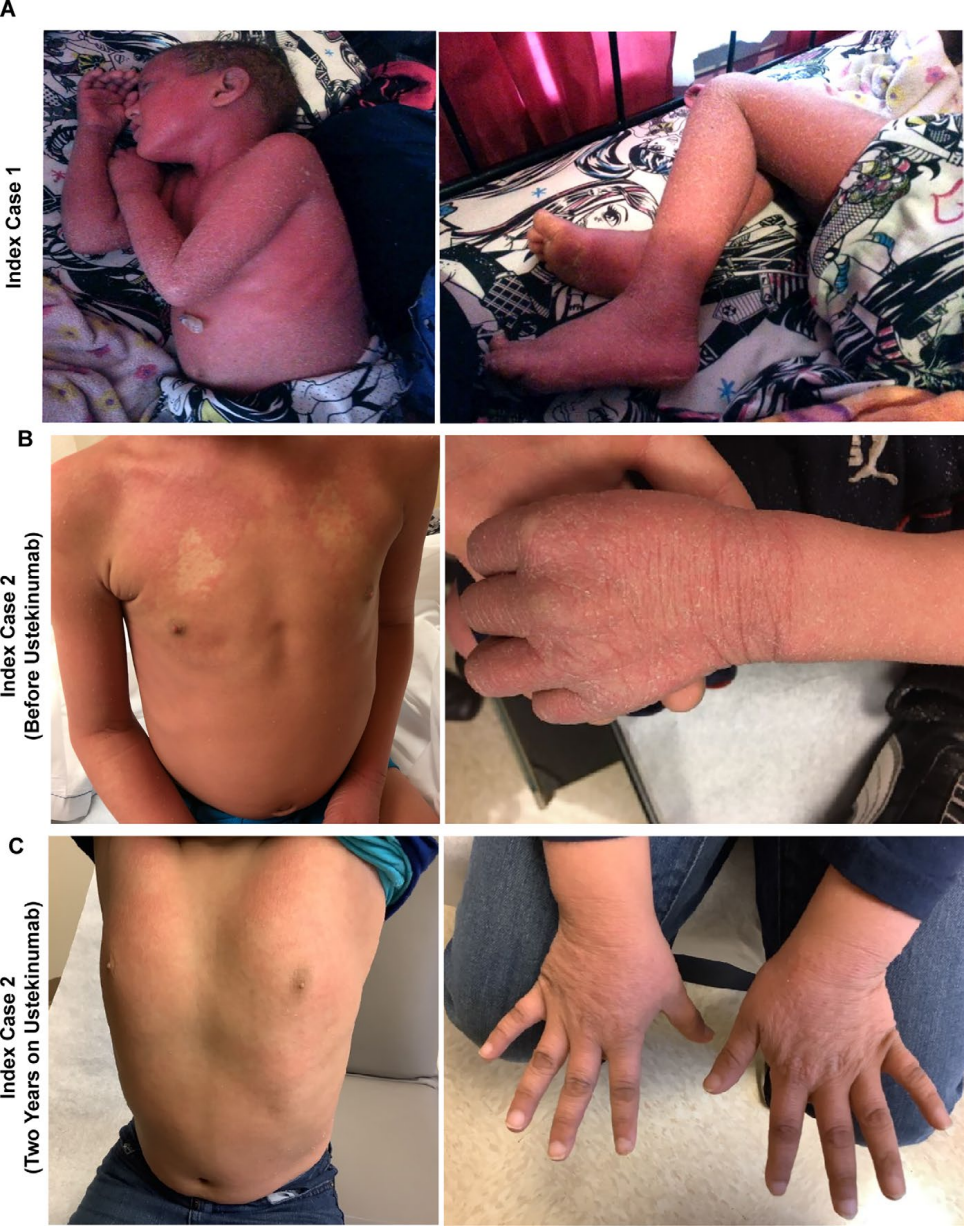
Photos of the patients showing generalized erythema with papules, pustules, and diffuse scaling: (A) Index case 1 and (B) Index case 2 before receiving usetikinumab, and (C) Index case 2 after 2 years of treatment with usetikinumab.

Index case 1 was an 8‐year‐old female with a history of generalized erythema with diffuse scaling of the face, trunk, and extremities since birth (Figure 1A). At 10 months of age, she developed superimposed papules, pustules, collarettes, and yellowish plates of scale. There was keratoderma on the palms and soles, sometimes accompanied by pustulosis. Her plantar feet showed diffuse pustulosis. Her scalp was affected by diffuse erythema and thick yellow scaling with alopecia in areas of thick scale. Her hair was wiry, coarse, dry, and thin. She was first seen by pediatric dermatology at the age of 2 years with a diagnosis of superinfected pustular psoriasis. No one else in the family had a similar skin condition. At that time, she also had swelling of the hands and feet with subungual hyperkeratosis of both fingernails and toenails. Dentition problems were noted, including several capped teeth and missing upper incisors. She developed cardiomyopathy and heart failure of unknown etiology. An echocardiogram at 2 years of age showed mild tricuspid regurgitation, trivial mitral regurgitation, normal left ventricular size, and systolic function. By age 8, the echocardiogram revealed mild mitral valve insufficiency, a mildly dilated left ventricle, mildly diminished left ventricular systolic function, and normal right ventricular cavity size and systolic function. She was placed on the heart transplant list but died at the age of 10.

Index case 1 had previously undergone genetic testing for *DOCK8*, *DIRA*, and *IL36RA* variants, all of which were negative. The patient underwent whole exome sequencing, which revealed that she was heterozygous for a missense variant in *DSP* (NM_004415.4):c.1769T>C (F590S). Sanger sequencing confirmed the variant was present in the proband and absent from the unaffected mother (Figure S1). The c.1769T>C (F590S) variant was not found at the time in dbSNP, Ensembl, 1000 Genomes, gnomAD Exomes, or gnomAD genomes. The in silico predictions showed a moderate to strong pathogenicity by multiple predictors (Table S1). The patient's father chose not to undergo testing, and he has never shown any manifestations related to EKC. Although we cannot completely exclude the possibility that the disease allele was inherited from him, we speculate that the mutation is de novo.

Index CASE 1 was treated with topical steroids, topical retinoids, calcipotriene, oral steroids, cyclosporine, subcutaneous methotrexate, acitretin, anakinra, adalimumab, etanercept, dilute bleach baths, chlorhexidine washes, systemic antibiotics, and oral antihistamines. All of these treatments produced limited or no response. She was never treated with ustekinumab.

### 2.2 Index Case 2

Index Case 2 had normal skin at birth, but developed a febrile illness at 3 months of age followed by a widespread pustular rash (Figure 1B). He had swelling of the hands and recurrent skin infections. Despite the rest of the family having dark hair and eyes, he had reddish‐blond hair and light‐colored eyes. At the time of presentation (5 years of age), he had low height and weight for his age, indicative of failure to thrive. He had generalized erythema with diffuse scaling of the face, trunk, and extremities, with superimposed papules and pustules. He had palmoplantar keratoderma. Echocardiogram showed normal heart function.

The patient's father chose not to undergo testing, and he has never shown any manifestations related to EKC. Although we cannot completely exclude the possibility that the disease allele was inherited from him, we speculate that the mutation is de novo. Index Case 2 underwent whole exome sequencing, which revealed that he was heterozygous for a missense variant in *DSP* (NM_004415.4):c.1768T>G (F590V). Sanger sequencing confirmed that the variant was de novo (present in the proband and absent from both parents' DNA, Figure S2). The variant was not found in dbSNP, Ensembl, 1000 Genomes, gnomAD Exomes, or gnomAD genomes.

Index Case 2 was treated with topical steroids, crisaborole 2%, and topical tacrolimus, all with limited response. After genetic diagnosis, the IL‐12/23 p40 inhibitor ustekinumab was administered subcutaneously at a dose of 0.75 mg/kg at weeks 0 and 4, followed by every 12 weeks after that. The patient had dramatic improvement in erythrokeratoderma and gain in height and weight by 6 months after therapy initiation (Figure 1C). He remains on ustekinumab and has never had cardiac disease.

**FIGURE 2:**
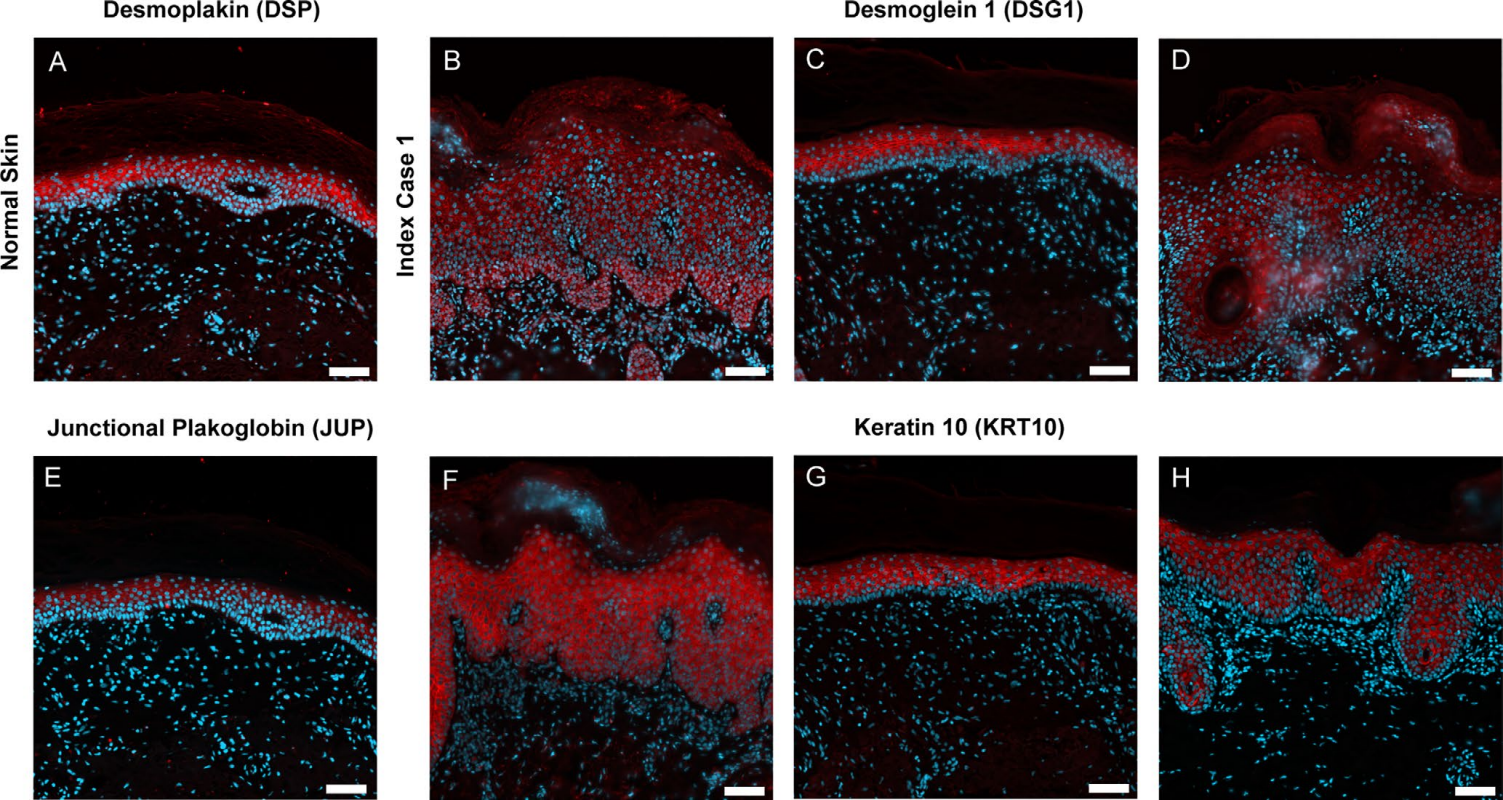
F590S EKC syndrome variant affects the expression of desmosomal proteins. Skin tissue from normal abdomen (top panels) and from the back of index case (bottom panels) were used for immunolocalization studies. DAPI nuclear counterstain is blue in each panel and all primary antibodies were detected by a secondary antibody shown in red. (A and B) Normal skin (A) shows prominent intercellular localization of desmoplakin (DSP) in suprabasal epidermis, while in affected tissue (B) intercellular localization is retained, but is more diffuse. (C and D) Desmoglein 1 (DSG1) localizes tightly to intercellular junctions of suprabasal keratinocytes of normal tissue (C), but in affected skin (D) intercellular staining of suprabasal cells appears less tightly focused. (E and F) Junctional plakoglobin (JUP) localizes to suprabasal keratinocyte cell membranes in normal skin (E), but in affected tissue (F) suprabasal intercellular staining intensity is diminished and is more diffuse. (G and H) Keratin 10 (KRT10) is strongly expressed and cytoplasmic in suprabasal cells of normal tissue (G) and affected skin (H). Scale bars = 50 μm.

To determine whether the F590S variant disrupted desmosomal proteins, we performed immunostaining on index case 1's skin and on normal skin (other donor) for the desmosomal proteins desmoplakin (DSP), desmoglein 1 (DSG1), junctional plakoglobin (JUP), and intermediate filament keratin 10 (KRT10). Although the F590S variant was associated with reduced overall signal intensity with more diffuse localization of the desmosomal proteins, it had no effect on the expression or localization of the intermediate filament keratin 10, consistent with results previously published on other EKC syndrome subjects (2) (Figure 2).

## 3 Discussion

Here, we address two cases of EKC due to heterozygous missense variants in the *DSP* gene causing substitution of the native amino acid phenylalanine at position 590 for serine and valine. Our results further suggest that the spectrum of EKC‐causing variants should be expanded beyond proline substitutions to include other damaging heterozygous variants in the SR6 domain.

The different clinical severities, specifically the cardiac manifestations, in two individuals with EKC caused by damaging variants at the same amino acid position (590) may be explained by the nature of the amino acid substitution. The F590S variant involves a non‐conservative substitution, replacing a hydrophobic phenylalanine (Phe) with a hydrophilic serine (Ser) within SR6 of the desmoplakin protein. In contrast, the F590V variant represents a conservative substitution, replacing phenylalanine (Phe) with another hydrophobic residue, valine (Val). This explanation is further supported by the presence of cardiac manifestations in a case of EKC caused by the F600S variant [5].

EKC patients have extensive ichthyosis, erythrokeratodermia, and elevated IL‐17 and IL‐22/IL‐23p40 levels, which may explain the improvement in symptoms with ustekinumab, an IL‐12/23 p40 inhibitor. EKC patients treated with ustekinumab can get both cutaneous and cardiac function improvement [5].

Taken together, these observations highlight the critical importance of early cardiac evaluation and the initiation of immune‐based therapies such as ustekinumab in EKC [5].

## Attestation

The authors attest that they are in receipt of consent from parents/guardians of each patient described for publication of the case history and images.

## Conflicts of Interest

K.A.C. has served as an investigator for Abbvie, Boehringer Ingelheim, and Regeneron. K.A.C. has received research funds from Jannsen. K.A.C. has served as a scientific advisor for Resvita and Biocryst.

## Supporting information


**Figure S1:** Chromatographs of the affected proband 1 (A) and the unaffected mother (B).

**Figure S2:** Chromatographs of the affected proband 2 and the unaffected parents confirm the de novo heterozygous variant described in this report.

**Table S1:** Pathogenicity of the F590S variant.
In silico prediction models for the pathogenicity of the F590S variant.

## Data Availability

The data that support the findings of this study are available from the corresponding author upon reasonable request.
