# Supplementary figures and images for: Erythrokeratodermia‐Cardiomyopathy Syndrome: Expanding the DSP Mutational Spectrum Beyond Proline Substitutions
Source: Pediatr Dermatol. 2025 Oct 14;43(2):444–7. doi: 10.1111/pde.70048 (PMC13051025; doi:10.1111/pde.70048)

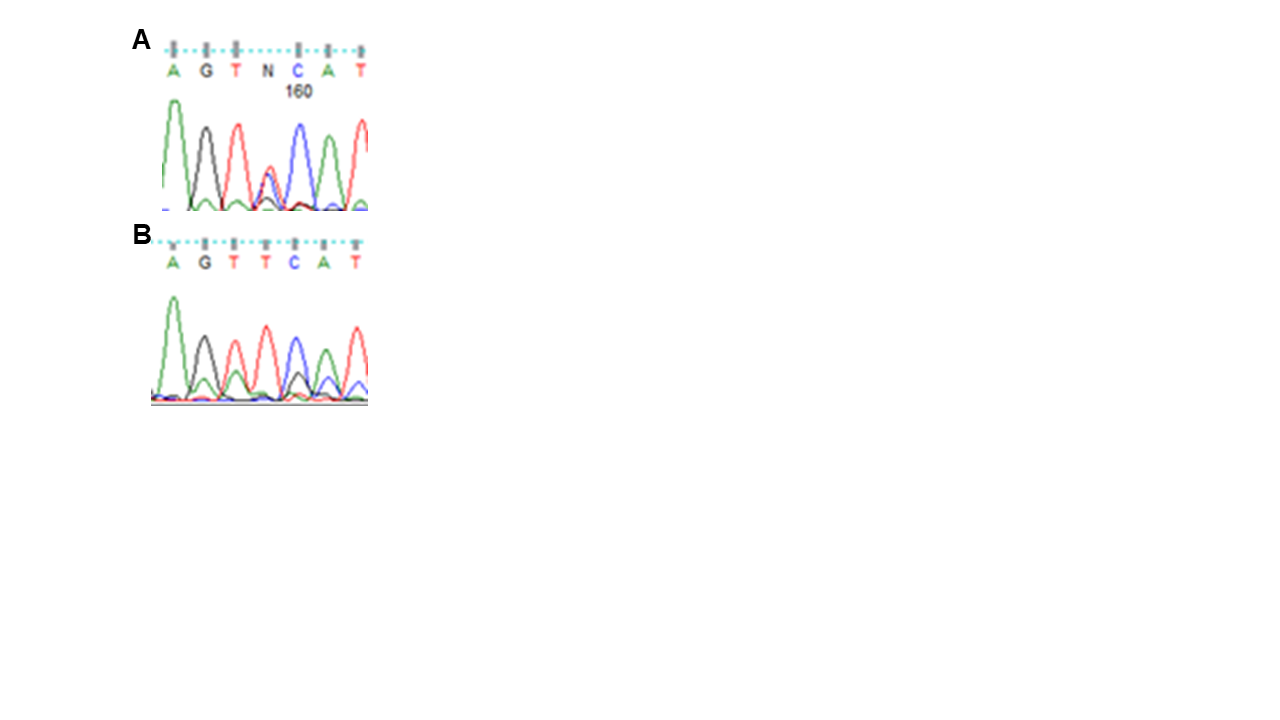

Supplement: Supplementary file 1 — Figure S1: Chromatographs of the affected proband 1 (A) and the unaffected mother (B). [file PDE-43-444-s001.tif]

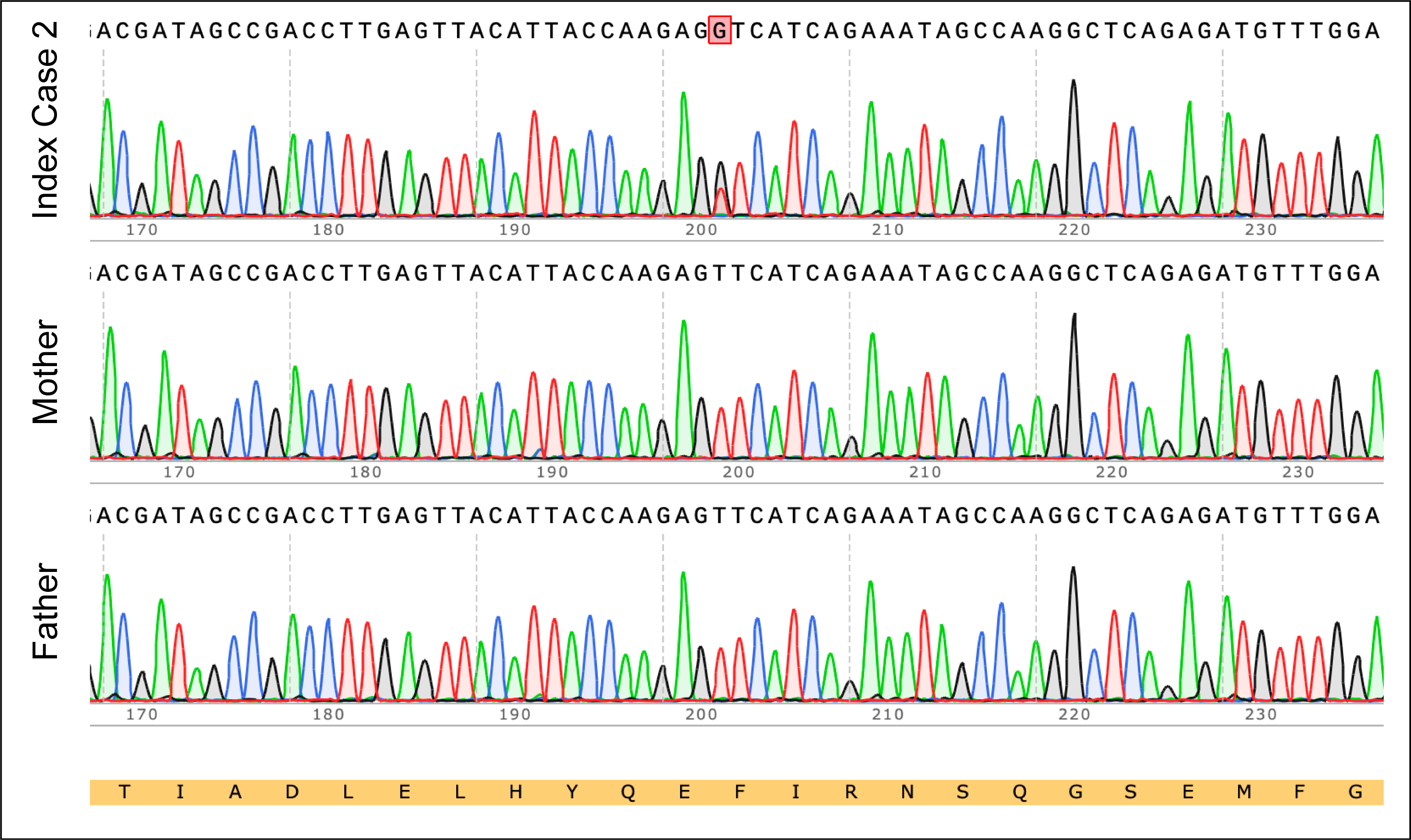

Supplement: Supplementary file 2 — Figure S2: Chromatographs of the affected proband 2 and the unaffected parents confirm the de novo heterozygous variant described in this report. [file PDE-43-444-s002.png]
