# Supplementary material for: Erythrokeratodermia‐Cardiomyopathy Syndrome: Expanding the DSP Mutational Spectrum Beyond Proline Substitutions
Source: Pediatr Dermatol. 2025 Oct 14;43(2):444–7. doi: 10.1111/pde.70048 (PMC13051025; doi:10.1111/pde.70048)
Supplement: Supplementary file 3 — Table S1: Pathogenicity of the F590S variant. In silico prediction models for the pathogenicity of the F590S variant. [file PDE-43-444-s003.docx]

| **Engine** | **Calibrated Prediction** | **Score** | **Indicative Prediction** |
| --- | --- | --- | --- |
| BayesDel addAF | Pathogenic Strong | 0.4507 | Damaging |
| BayesDel noAF | Pathogenic Moderate | 0.4097 | Damaging |
| MetaRNN | Pathogenic Moderate | 0.8816, 0.8816 | Damaging, Damaging |
| REVEL | Pathogenic Moderate | 0.931, 0.931 | Damaging, Damaging |
| DEOGEN2 | Pathogenic Moderate | 0.9174 | Damaging |
| EIGEN PC | Pathogenic Moderate | 0.8063 | Damaging |
| FATHMM-XF | Pathogenic Moderate | 0.9674 | Damaging |
| MVP | Pathogenic Moderate | 0.9751, 0.9751 | Damaging, Damaging |
| PROVEAN | Pathogenic Moderate | -7.18, -5.4 | Damaging, Damaging |
| SIFT | Pathogenic Supporting | 0, 0 | Damaging, Damaging |
